# Nutritional Status of Saudi Children with Celiac Disease Following the Ministry of Health’s Gluten-Free Diet Program

**DOI:** 10.3390/nu14142792

**Published:** 2022-07-07

**Authors:** Shiekhah S. Allowaymi, Manal Abdulaziz Binobead, Ghedeir M. Alshammari, Ali Alrasheed, Mohammed A. Mohammed, Mohammed Abdo Yahya

**Affiliations:** 1 Department of Food Science and Nutrition, College of Food and Agricultural Sciences, King Saud University, Riyadh 11451, Saudi Arabia; 439203981@student.ksu.edu.sa (S.S.A.); mbinobead@ksu.edu.sa (M.A.B.); muhammed-awad@hotmail.com (M.A.M.); mabdo@ksu.edu.sa (M.A.Y.); 2 General Administration of Nutrition, Ministry of Health, Riyadh 11345, Saudi Arabia; alfalrasheed@moh.gov.sa

**Keywords:** children, celiac, dietary intake, body mass index, body fat

## Abstract

This study aimed to evaluate the nutritional status of Saudi children with celiac disease (CD) who followed the Ministry of Health’s gluten-free diet (GFD) program. This study involved 66 children with CD (29 boys and 37 girls) from 5 hospitals belonging to the Ministry of Health. Socioeconomic characteristics were obtained using a structured questionnaire. Anthropometric indices were measured using a body composition analyzer. Dietary intake was assessed using three 24 h dietary records. The biochemical parameters were determined in the hospitals’ laboratories. According to the findings, the majority of respondents had ages ranging from 10 to 13 years, a father and mother with a university education, a high family income, and 5 to 7 family members. Carbohydrates and protein intake for both genders were significantly higher than the DRI’s recommended dietary intake. However, the majority of nutrients consumed were at levels significantly lower than the DRI. Both genders had normal anthropometric indices, with girls having at significantly higher indices than boys. The biochemical parameters of both genders were comparable and within the normal range, except for vitamin D, which was below the normal range. The most important factors influencing nutritional status were age for both genders, and family income and number of family members for boys. In conclusion, data obtained for nutrient intake, anthropometric indicators, body composition, and biochemical analysis indicated that CD children following the Ministry of Health GFD program have a generally good nutritional status.

## 1. Introduction

Gastrointestinal diseases include irritable bowel syndrome, inflammatory bowel disease (IBD), celiac disease (CD), gastrointestinal cancer, and esophageal reflux [1]. CD is chronic autoimmune disease enteritis caused by an immune reaction to gluten, which then causes the small intestine to present villi atrophy and malabsorption [2]. It has been more commonly studied in recent years due to its prevalence, its diagnosis with new tools, and the comprehensive screening of individuals considered to be at risk for CD [3]. CD has recently been identified as a common worldwide disease affecting children and adults [4].

CD is a chronic immune disorder that occurs after ingestion of gluten among genetically predisposed people, which causes progressive atrophy of the villi in the small intestine [5]. Gluten is a protein complex in grains such as wheat, rye, barley, or any products to which gluten is added [6]. CD prevalence is about 1% worldwide, with slight differences between countries [3]. Currently, CD is considered a high-risk disease, especially in children with CD, due to many factors, such as the malabsorption of nutrients due to the need to follow a lifelong GFD, along with the limited availability and high cost of such dietary requirements [4]. Such factors may lead to a negative impact on growth and nutritional status [7].

Symptoms of CD often occur in the gastrointestinal system, such as nausea, vomiting, diarrhea, constipation, abdominal pain, and abdominal distention [8]. A major complication of CD is malabsorption caused by villi damage in the small intestine, which leads to nutritional deficiencies that involve a lack of macro-and micronutrients [9]. As a result, a lack of nutrients leads to other symptoms that may impair growth and cause health issues, such as anemia, weight loss, depression, increased mortality rate, abnormal growth, osteoporosis, tooth enamel defects, delayed puberty, and dermatitis herpetiform [10].

Currently, the only treatment available for CD is the lifelong GFD, which consists of the dietary exclusion of gluten [11]. Adherence to GFD is a difficult challenge for celiac patients of all ages, particularly for children and adolescents [12]. Following GFD mean the exclusion of several foods in dietary habits; such exclusion may lead to deficiency in some nutrients, affecting patients’ nutritional status [13]. Studies on patients with CD after a long time of adhering to a GFD showed a lower intake of energy and other nutrients than recommended, and patients were advised to take in more fats, proteins, and simple carbohydrates [14].

In Saudi Arabia, several studies found a prevalence of CD ranging between 1–4%, which makes Saudi Arabia one of the countries with a high prevalence of CD [15]. Sarkhy et al. [2] expected a high prevalence rate of CD among children in Saudi Arabia. Therefore, in May 2018, the Saudi Ministry of Health approved a program providing a gluten-free diet for celiac patients by the General Nutrition Administration. The number of registered patients in the program throughout the country was 2608 patients. The gluten-free diet is distributed monthly through 33 hospitals across the country. In the Riyadh region, the number of registered CD patients in the program reached 976, including approximately 98 children. Therefore, this study has been conducted to evaluate the nutritional status of Saudi children with celiac disease (CD) following the Ministry of Health’s gluten-free diet program.

## 2. Materials and Methods

### 2.1. Sample Size and Participants

The sample size was calculated based on a database of the General Nutrition Department at the Saudi Ministry of Health, where 98 children were registered in the Ministry of Health for the GFD program in Riyadh city. The samples (both genders) with an age range from 6 to 16 years were selected, using the convenience sampling technique, from 5 hospitals belonging to the Ministry of Health that provided the GFD. All patients had previously tested positive for the anti-tissue transglutaminase IgA class antibodies (anti-tTG antibodies), diagnosed with CD, and registered in the GFD program for more than 6 months. All selected CD patients (66 out of 98) followed a GFD and did not have cancer, type 1 diabetes, food allergies (except gluten), hepatitis C, kidney failure, or follow a special diet for any reason. Only 66 participants met the inclusion criteria; other participants were excluded, and the selected patients were divided into two groups—boys and girls.

### 2.2. Data Collection

The data were collected using a structural questionnaire, approved by an expert committee, from January to December 2021 from 5 hospitals, including Al-Iman General Hospital, King Salman Hospital, King Saud Medical City, King Fahad Medical City, and Prince Mohammed Bin Abdulaziz Hospital. The researcher interviewed the patients and their parents via a previously scheduled appointment at the clinic, which was coordinated with the nutrition department at each hospital. One of the parents signed written informed consent before starting the study. Demographic data of the children and their families were collected using a structured questionnaire approved by specialists, and the questionnaire involved questions about age, age at diagnosis, gender, the income of the family, number of family members, and father’s and mother’s education.

### 2.3. Dietary Intake and Analysis

The patients’ 24 h food intake was tracked using an approved structured questionnaire for the previous three days, two weekdays and one weekend day. The dietary intake included a daily record of all foods consumed during the day, including breakfast, lunch, dinner, and snacks. The patients and their parents had previously received instructions from the researcher and were requested to report an accurate description of food brand name, food amount—using the common measures such as a tablespoon, teaspoon, cup, or size by gram or liter of packaged food—preparation method, and food additives used during cooking. After completion, they were requested to send the description via WhatsApp and were later contacted to clarify all details to ensure that all data were complete. The dietary intake analysis was determined using a nutrition analyzer software program designed for research and clinical purposes (ESHA Food Processor 11.9.13, 2020, ESHA Research, Salem, OR, USA). This software gives a code for each type of food, whether fresh or cooked. Then three 24 h dietary records for each patient were entered, e.g., one cup milk, two slices of gluten-free toast with fried egg, 100 g gluten-free pasta, cooked, 60 g green salad, etc. After that, the program analyzed the food entered and provided the nutrients, such as calories, carbohydrates, protein, etc. The nutrients obtained were compared with the program for dietary reference intake [16].

### 2.4. Anthropometric Measurements

All anthropometric and body composition data for the children, including weight (kg), height (cm), body mass index (BMI, kg/m^2^), fat mass (FM), fat-free mass (FFM), and muscle mass (MM) (kg and %), were measured by the researcher, using a body composition analyzer (model number MC-780MA, TANITA Corp., Tokyo, Japan) and a stadiometer for height, with light clothes, socks, and shoes removed. BMI-for-age and height-for-age were determined by plotting the z-score using the national growth chart for Saudi children and adolescents [17]. The classification was a short stature as ˂−2 standard deviation (SD), overweight as BMI ˃ +1 SD, obesity as BMI ˃ +2 SD, and thinness as BMI ˂ −2 SD [18].

### 2.5. Biochemical Parameters

At the patients’ hospital laboratory, biochemical parameters, such as vitamin D (nmol/L), hemoglobin (g/dL), albumin (g/L), sodium (mmol/L), potassium (mmol/L), alkaline phosphatase (U/L), and calcium (mmol/L), were measured. The machine manufacturer’s manual was used to obtain the reference ranges.

### 2.6. Statistical Analysis

The data were analyzed using the Statistical Package for the Social Sciences (SPSS, version 26). Categorical variables were described as a frequency and percent, and continuous variables were described as a mean ± standard deviation (SD). A one-sample t-test was used to compare the means of nutrient intake of children to the dietary reference intake (DRI). An independent sample t-test was used to compare the means of biochemical parameters between genders, as well as to compare body composition. The chi-square test was used to compare categorical variables between genders. To determine the correlation between anthropometric indices as a dependent variable and socioeconomic characteristics as independent variables, Spearman correlation coefficients and simple linear regression analysis were used. Values less than or equal to 0.05 were considered statistically significant.

### 2.7. Ethical Considerations

This study was multicenter-approved by: King Saud University, Subcommittee on the Ethics of Human and Social Research, Ref No. KSU-HE-20-633; the Ministry of Health, Riyadh, First Health Cluster by the Institutional Review Board Committee at King Saud Medical City, Ref No. H1RI-29-Jun21-01; and the Ministry of Health, Riyadh, Second Health Cluster by the Institutional Review Board Committee at King Fahad Medical City, Ref No. 21-209E.

## 3. Results

### 3.1. Demographic Characteristics

Table 1 shows the frequency distribution of children with CD following the Ministry of Health GFD program for both genders, according to demographic characteristics. The participants with CD were 29 boys and 37 girls, ranging from 6 to 16 years old. The frequency distribution according to age showed that most children with CD in both genders fell within the age range of 10 to 13 years (44.8% for boys and 45.9% for girls). The lowest percent of CD frequency was observed for boys 14 to 16 years (20.7%), while that of girls was from 6 to 9 years (16.2%). With a few exceptions, the age at diagnosis for both genders was comparable.

The parents of both groups had either a secondary school or university education, with the majority having a diploma or university certificate, followed an intermediate or secondary school education. The majority of boys’ fathers (62.1%) had a diploma or university education, followed an intermediate or secondary school education (27.6%), while girls’ fathers (40.6%) had an intermediate or secondary education, followed by a diploma or university certificate (37.8%). Boys’ and girls’ mothers had a diploma or university education (41.4% and 45.9%, respectively), followed by an intermediate or secondary school education (37.9%, 27%). The percentage of fathers and mothers who obtained a postgraduate degree was low. However, for girls, the percentage of fathers who obtained a postgraduate degree was higher than that of mothers, and parents of girls who obtained a postgraduate degree outnumbered parents boys with such degrees. Furthermore, for both sexes, the percentage of illiterate fathers was lower than that of illiterate mothers.

The results showed that monthly income for most families in both groups was ≥SAR 16,000 (37.9% for boys and 27.0% for girls), followed by intermediate income (SAR 6000–10,000) for boys (31%), and low income for girls’ family (SAR 2000–5000). The number of family members for both groups’ families ranged from 5 to 7 members (41.4% for boys and 64.9% for girls), followed by members ranging from 8 to 10, with a few members ˃ 10. Family members of 2 to 4 were more common for boys’ family (24.1%) compared to girls’ (8.1%).

### 3.2. Dietary Intake

To determine the average nutrient intake according to food consumed by the children, the ESHA program was used, and the children’s nutrient intake was then compared with the average dietary reference intake (DRI) of each nutrient. Using a Student t-test, the mean of each constituent was compared with the mean of the dietary requirement intake. Table 2 summarizes the average intake of nutrients (three 24 h dietary records) compared to the dietary reference intake (DRI) of children with CD following the Ministry of Health GFD program. The results showed that both genders’ average intake of calories and macronutrients was significantly different (*p* ≤ 0.01) compared to the DRI, except for fat, and some vitamins and minerals. Calories and fiber were significantly lower than the DRI. Protein and carbohydrates were significantly (*p* ≤ 0.01) higher than the DRI, but no significant difference in fat intake was noted for either gender. In the case of vitamins, most dietary intakes of vitamins were significantly lower (*p* ≤ 0.01, *p* ≤ 0.05) than the DRI, including vitamins A, B1, B6, D, E, and folate. However, for girls, the intake of vitamins B2, B3, and C were significantly lower than for boys, while vitamin K was significantly lower in boys than in girls, except for vitamin B12, which was consumed above the DRI by both genders. All mineral intake was inadequate in both genders; it was significantly lower (*p* ≤ 0.01) than the DRI, except for iron, which was significantly lower in girls than in boys, and sodium was not significantly low in either gender.

### 3.3. Anthropometrics Measurements

The BMI-for-age, height-for-age, fat mass (%), and muscle mass (%) of the children with CD following the Ministry of Health GFD program were measured. The BMI-for-age and height-for-age were classified according to the Saudi national growth chart z-score standard deviation (SD). As shown in Table 3 and according to the chi-square test, there was a significant (*p* ≤ 0.01) difference in classification degree for both genders regarding BMI-for-age and height-for-age. For BMI-for-age, the majority of children were normal (89.7% for boys and 67.6% for girls). Among children, the overweight and thinness characteristics, with a low percentage difference between genders, showed a higher percentage for girls than boys. Obesity was not common among boys, with only one case in girls. Additionally, height-for-age for both genders generally was normal (86.2% for boys and 97.3% for girls). Short stature was uncommon, but was more common in boys than in girls (13.8%, 2.7%, respectively).

The FM and MM% for the children with CD, according to the body composition analyzer, were varied between genders. According to the chi-square results, there was a significant (*p* ≤ 0.01) difference in classification level for both genders regarding FM and MM. Both genders showed high rates of the normal level of FM (65.5%, for boys and 54.1% for girls), followed by a high level (27.6% for boys and 43.2% for girls), with an increased percent for girls, followed by a low level. The majority of respondents had normal MM (62.1% for boys and 51.4% for girls), followed by a low level (31.0% for boys and 45.9% for girls), with an increased percentage in girls. As shown in Table 4, a comparison of anthropometric measurements and body composition between boys and girls showed that girls had significantly (*p* ≤ 0.05, ≤0.01) higher mean values for all anthropometric measurements, except for FMM, MM, and SMM, which were significantly (*p* ≤ 0.01) higher in boys than girls.

### 3.4. Biochemical Parameters

Table 5 shows the data for biochemical parameters of CD children. According to the independent t-test, there was no significant difference in all parameters, except vitamin D and potassium, which differed significantly (*p* ≤ 0.05) between boys and girls. All parameters were within the normal range in both genders, with the exception of vitamin D levels, which were below the normal range.

### 3.5. Factors Associated with Nutritional Status of Children

Table 6 summarizes the Spearman correlation and simple linear regression analysis performed on the demographic characteristics of the CD children as independent variables, and anthropometric indices as dependent variables. The independent variables were found to be positively or negatively correlated with the anthropometric indices (BMI, FM, and MM). According to simple regression analysis, the age of CD children was positively and significantly correlated (*p* ≤ 0.01) with all anthropometric indices, with a high effect observed on MM for both genders. The results for parents’ education showed a negative correlation with all anthropometric indices for both genders, but the correlation was not significant. Monthly income was positively and significantly (*p* ≤ 0.05) correlated with anthropometric indices in boys. The number of family members, on the other hand, had a positive and significant (*p* ≤ 0.05) correlation with FM and MM in boys, but did not affect anthropometric indices in girls.

## 4. Discussion

The purpose of this study was to investigate the nutritional status of Saudi children with CD following the Ministry of Health’s GFD program in Riyadh city. CD is two to five times more common in children than in adults, and children are more likely to exhibit the classical CD, with gastrointestinal symptoms, whereas adults are more likely to have non-classical CD [19]. According to the demographic characteristics, the education level of the children’s parents was either secondary school or university education, and the percentage of illiterate fathers was lower than that of illiterate mothers. However, the difference in the effects of maternal and paternal education is especially likely to be biased upwards in absolute value. One of the most important factors determining children’s nutritional status is their parents’ education. The education of parents, as well as mothers and fathers separately, has a direct relationship with nutrition; that is, as educational standards improve, children’s nutritional status improves. This could be due to educated parents playing a larger role in pursuing the appropriate strategy for making a larger share of household resources available to children [20]. The education of mothers appears to be an important factor in children’s nutrition status. There is a consistent and strong link between increased maternal education and child survival and development [21]. An educated mother likely brings up her child in a better way than an uneducated one. Father’s education is necessary for the sound nutritional status of the child. Essential care for the children also depends on the father’s education level because the main household income earners in Saudi Arabia are predominantly males [22]. For the majority of the children’s families, the income was sufficient to meet their needs.

The findings revealed that the families under investigation had more family members, which could be attributed to the fact that the parents’ economic situation in Saudi Arabia is stable, as well as the general environment, which encourages parents to have more members. According to the research of Fentaw et al. [23], the majority of undernourished children have more than five family members. Poor allocation of household resources makes it impossible to meet the nutritional needs of all members equally. Children in large families, in particular, have limited access to adequate and nutritious food. Another study conducted by Ajao et al. [24] found that children in large families had lower food availability than children in small families.

According to children’s dietary intake, both genders consumed more protein, carbohydrates, a reasonable amount of fat, and some vitamins (B2 for boys), but consumed fewer other nutrients than the DRI. According to the study by Kozioł-Kozakowska et al. [25], celiac children consumed few calories and did not meet the dietary requirements after one year of the GFD. Similarly, it has been reported that children with CD consume more protein than is recommended, owing primarily to the consumption of animal protein foods [26]. The high carbohydrate intake by the children with celiac under investigation is largely dependent on the GFD provided by the Saudi Ministry of Health, which includes flour, bread, pasta, oats, crackers, and cornflakes.

Furthermore, the main dish for lunch and dinner was rice with meat and GF pasta or macaroni, which are high in carbohydrates. Additionally, a review concluded that GF products might have a higher content of carbohydrates than gluten-containing products [13]. Fiber intake for both groups falls short of the recommended level, consistent with a study conducted in Spain on 70 children and adolescents with CD, which found that fiber intake was less than 25 g per day [4]. Low fiber intake, as well as a low intake of some vitamins and minerals in CD children, is primarily due to the low intake of vegetables and fruits [27], and a GFD was low in fiber because of flour refining [14].

According to a German study, both genders with CD had significantly lower average micronutrient intakes than the general population, particularly vitamins B1, B2, B6, and folate [14]. The lack of such nutrients could be attributed to the consumption of a GFD, which contains significantly less vitamin D, E, B12, iron, folate, magnesium, potassium, and sodium than gluten-containing products [28]. Furthermore, it should be noted that the lack of exposure to sunlight, rather than diet, is the primary cause of vitamin D deficiency [25]. A study conducted in Jeddah, Saudi Arabia, to assess the prevalence of vitamin D deficiency among Saudi children revealed a 54.9% prevalence [29]. Studies among Saudi children indicated inadequate intake of minerals and nutrients [27,30]. Mineral deficiency in celiac patients could be caused by an insufficient amount of minerals in the GFD [31]. A recent study on celiac children found no significant increase in iron intake for children who followed the GFD for a year [25]. In keeping with the fact that people with celiac disease frequently develop anemia, the children under investigation were not given iron supplements, so the results reflect their actual iron intake.

The results of BMI-for-age, height-for-age, FM%, and MM% showed that the majority of children were normal, with few exceptions. These findings are consistent with those reported by Fernández et al. [4], who found that the majority of the children had normal BMI, with only a small percentage being overweight or obese. Sansotta et al. [32] also conducted a study in two countries that included 125 Italian and 140 American children with CD who followed a GFD for more than 6 months and observed an evaluated BMI-for-age z-score and percentiles for Italian and American children. In contrast, Moya et al. [33] found that BMI z-score and height z-score did not change significantly after one year of diagnosis, which could be attributed to poor adherence to the GFD. According to these findings, the present results support that adherence to a GFD will enhance the absorption of nutrients and thus improve growth and nutritional status [34].

The percentage of FM and MM in children varied between the sexes, with girls having a high percentage of FM and boys having a high percentage of MM. The present results were consistent with previous studies in Mexico and Poland that measured the body composition of girls and boys and found a tendency to increase FM% in girls and MM% in boys [35,36]. The current results of children’s body composition confirmed that, as part of their physiology, girls naturally store more fat than boys [37].

When anthropometric indices of boys were compared to those of girls, the majority of indices for girls were significantly higher than those for boys, with the exception of FFM, MM, and SMM percentages, which were significantly higher in boys. The current results were in agreement with a previous study conducted in Croatia among 1149 boys and girls that compared body composition between genders, which showed that girls had significantly higher FM, FM, and FMI than boys and significantly lower FFM than boys [38]. Moreover, a study showed that the percentage of FM was higher in Czech girls than boys, and FFM (kg) and SMM (kg) were higher in boys than girls [39].

In contrast to the current study, González-Ruz et al. [40] conducted a study with 42 boys and 85 girls in Latin America and discovered no significant differences in anthropometric characteristics between boys and girls. Furthermore, a study of 35 celiac children and adolescents in Spain discovered no gender differences in anthropometric measurements such as weight, height, and BMI [4]. In addition, Al-Hazzaa et al. [41] found no significant differences in anthropometric measurements between boys and girls in a study of 1149 children and adolescents from schools in Jeddah, Saudi Arabia. The difference between this study and previous studies could be attributed to the age distribution of boys and girls, as well as the number of participants, which revealed that girls outnumbered boys.

The biochemical parameters were within the normal range, and no significant differences between genders were observed except for vitamin D, which was higher in girls than boys, but still significantly lower than the reference range for both genders. The current findings agreed with a previous study conducted in Spain on 70 children and adolescents on a GFD for a long period, which found that biochemical values in CD children were within normal ranges, with no statistical difference between genders, except for vitamin D levels, which were below normal reference values [4]. Furthermore, a study conducted in Canada on 140 CD children of both genders 6 months after beginning a GFD revealed that the common deficiency among them was vitamin D [42]. A variety of factors cause vitamin D deficiency. Vitamin D3 is primarily produced in the skin by converting 7-dehydrocholesterol under the influence of ultraviolet light B (UVB) from the sun or other UVB sources [43].

A correlation between CD children’ demographic characteristics as independent variables and anthropometric indices as dependent variables was carried out using the Spearman correlation and simple linear regression analysis. A significant and positive correlation of age with BMI, FM, and MM was found to be natural; as age increases, so should anthropometric measurements, as indicated by AlZoubi et al. [44], who observed a positive correlation between age and BMI for Syrian children in Saudi Arabia. Furthermore, age and FM were found to have a significant and positive correlation among Saudi students [45]. In addition, a study among 2182 Chilean children showed a positive and significant correlation between age and MM [46]. Regarding family monthly income, a positive and significant correlation was observed in boys only, due to increasing the percentage of income in the high category SAR ≥ 16,000 (37.9%) compared to girls’ family income. High income enables families to obtain high-quality foods, resulting in an increase in anthropometric measurements. However, Alazzeh et al. [47] discovered a negative relationship between family income and obesity in male students from the Hail region of Saudi Arabia. This could be due to the limited food resources available to low-income families. Previous research on foreign students in Saudi Arabia (44, 45) obtained similar results. The findings for the number of family members being positively and significantly correlated with boys’ FM and MM contradicted the findings of a study on celiac patients in Pakistan, which found no correlation between the number of family members and anthropometric measurements [48]. Moreover, Ajao et al. [24] discovered that children in large families had lower food availability than children in small families. However, a recent study in Jeddah, Saudi Arabia, found that children from families with 4–6 members or more tended to have higher anthropometric measurements than those with 3 or fewer family members [49]. According to our study, socioeconomic characteristics represent a major factor favoring the wellbeing of children. Therefore, a detailed future study on the impacts of socioeconomic characteristics is required. Additionally, a study on the importance of early diagnosis and the early introduction of a GFD to improve the CD children’s nutritional status is recommended, as well as to evaluate of the effect of a GFD on clinical manifestations and histologic findings regarding CD children in Saudi Arabia.

## 5. Conclusions

The study focused on the nutritional status of children with CD who had been following the Ministry of Health’s strict GFD for a long time, which was expected to contain a large number of restrictions that could affect the children’s quality of life and nutritional status. According to the data, children with CD who followed a strict GFD for more than 6 months generally had a good nutritional status. The socioeconomic characteristics were varied between respondents. In the present study, a deficit in some nutrients when comparing CD children’s nutrient intake to the DRI was observed. However, the protein, carbohydrates, and fat intake were adequate. Moreover, the majority of the children had normal anthropometric measurements. Except for vitamin D, children’s biochemical parameter values were within the reference range. Boys’ and girls’ ages favor nutritional status, as evidenced by a positive correlation with anthropometric indices. Furthermore, family income and size had a positive effect on the nutritional status of boys. These findings support the need for personalized nutritional education that focuses not only on eliminating gluten from the diet, but also on reducing nutritional deficiencies by selecting nutrient-dense foods when a diet deficiency is detected to improve patient well-being.

## 6. Study Limitation

The current study looks at dietary intake and body composition. However, there are some limitations, such as that a portion of the study was cross-sectional, so the results should be interpreted cautiously. The sample size was low due to the small number of available cases, since this study focuses on a specific region in Saudi Arabia, and due to the size of the country, it is difficult to cover the entire country with one study. Furthermore, the number of patients was greater than 66, but only 66 met the criteria for the study.

## Figures and Tables

**Table 1:** Distribution of the frequency of children (*n* = 66) with CDs following the Ministry of Health GFD program, according to demographic characteristics.

Variables	Boys (*n* = 29)		Girls (*n* = 37)	
	Frequency	%	Frequency	%
Age (years)				
6–9	10	34.5	6	16.2
10–13	13	44.8	17	45.9
14–16	6	20.7	14	37.9
Age at diagnosis (years)				
2–6	11	37.9	10	27.0
7–11	15	51.7	14	37.8
12–16	3	10.4	13	35.2
Fathers’ education				
Illiterate/Primary	2	6.9	2	5.4
Secondary Intermediate	8	27.6	15	40.6
Diploma/University	18	62.1	14	37.8
Postgraduate	1	3.4	6	16.2
Mothers’ education				
Illiterate/Primary	4	13.8	7	18.9
Secondary/Intermediate	11	37.9	10	27.0
Diploma/University	12	41.4	17	45.9
Postgraduate	2	6.9	3	8.2
Family’s monthly income				
SAR 2000–5000	5	17.3	10	27.0
SAR 6000–10,000	9	31.0	8	21.6
SAR 11,000–15,000	4	13.8	9	24.4
≥SAR 16,000	11	37.9	10	27.0
Number of family members				
2–4	7	24.1	3	8.1
5–7	12	41.4	24	64.9
8–10	8	27.6	8	21.6
10˃	2	6.9	2	5.4

**Table 2:** The average nutrient intake in relation to dietary reference intakes (DRI) for children with CD following the Ministry of Health GFD program.

Items Intake	DRI	Boys				Girls			
		Intake (Mean)	Difference	*t*-Test	*p*-Value	Intake (Mean)	Difference	*t*-Test	*p*-Value
Calories (kcal)	1800	1400.18	−399.82	−4.80 **	0.000	1296.15	−303.85	−4.43 **	0.000
Protein (g)	34	47.37	13.37	3.53 **	0.001	42.94	8.93	4.80 **	0.000
Carbohydrates (g)	130	192.86	62.86	6.22 **	0.000	172.44	42.44	4.21 **	0.000
Fiber (g)	25	12.85	−12.15	−10.4 8 **	0.000	12.51	−9.48	−8.89 **	0.000
Fat (% kcal)	30	32.05	2.05	1.83	0.121	34.48	4.48	5.91	0.213
Vitamin A, RAE (mcg)	600	326.16	−273.84	−3.12 **	0.004	247.09	−352.90	−9.05 **	0.000
Vitamin B1 (mg)	0.9	0.69	−0.21	−2.42 *	0.022	0.53	−0.37	−8.62 **	0.000
Vitamin B2 (mg)	0.9	0.94	0.03	0.41	0.683	0.72	−0.18	−3.06 **	0.004
Vitamin B3 (mg)	12	9.62	−2.37	−1.94	0.062	7.97	−4.03	−5.84 **	0.000
Vitamin B6 (mg)	1.0	0.66	−0.34	−4.93 **	0.000	0.54	0.46	−9.38 **	0.000
Vitamin B12 (mcg)	1.8	2.54	0.74	0.85	0.403	1.82	0.01	0.04	0.967
Vitamin C (mg)	45	36.18	−8.82	−1.68	0.104	32.24	−12.76	−3.18 *	0.003
Vitamin D (mcg)	15	3.26	−11.74	−28.86 **	0.000	2.50	−12.50	−41.04 **	0.000
Vitamin E (mg)	11	1.45	−9.55	−61.66 **	0.000	1.59	−9.41	−44.38 **	0.000
Folate, DFE (mcg)	300	142.09	−157.91	−11.71 **	0.000	146.52	−153.48	−10.60 **	0.000
Vitamin K (mcg)	60	34.71	−25.28	−2.45 *	0.021	62.25	2.25	0.17	0.865
Calcium (mg)	1300	531.45	−768.55	−16.80 **	0.000	496.58	−803.41	−25.47 **	0.000
Iron (mg)	8	7.68	−0.32	−0.44	0.666	6.22	−1.78	−4.19 **	0.000
Magnesium (mg)	240	97.85	−142.14	−15.79 **	0.000	94.64	−145.35	−17.50 **	0.000
Phosphorus (mg)	1250	494.23	−755.76	−19.10 **	0.000	458.84	−791.16	−19.08 **	0.000
Potassium (mg)	2500	1175.18	−1324.82	−12.29 **	0.000	1032.87	−1267.13	−16.97 **	0.000
Sodium (mg)	1800	1705.44	−94.56	−0.76	0.455	1666.19	−133.81	−0.70	0.486
Zinc (mg)	8	3.37	−4.63	−17.96 **	0.000	3.15	−4.85	−19.52 **	0.000

*t*-test: * *p* ≤ 0.05; ** *p* ≤ 0.01; difference = mean DRI.

**Table 3:** BMI-for-age, height-for-age, fat mass, and muscle mass of children (*n* = 66) with CD following the Ministry of Health GFD program.

Anthropometric Index	Boys (*n* = 29)		Girls (*n* = 37)		Total (*n* = 66)	
	Frequency	%	Frequency	%	Frequency	%
BMI-for-age						
Normal	26	89.7	25	67.6	51	77.3
Overweight	2	6.9	7	18.9	9	13.6
Obesity	0	0	1	2.7	1	1.5
Thinness	1	3.4	4	10.8	5	7.6
Height-for-age						
Normal	25	86.2	36	97.3	61	92.4
Short stature	4	13.8	1	2.7	5	7.6
Fat Mass (%)						
Low	2	6.9	1	2.7	3	4.5
Normal	19	65.5	20	54.1	39	59.1
High	8	27.6	16	43.2	24	36.4
Muscle Mass (%)						
Low	9	31.0	17	45.9	26	39.4
Normal	18	62.1	19	51.4	37	56.1
High	2	6.9	1	2.7	3	4.5

Height-for-age defined by normal (˂−1–˃+2 SD), short stature (˂−2 SD); chi-square = 47.51. BMI-for-age defined by normal (˂−1–˂+1 SD), overweight (˃+1 SD), obesity (˃+2 SD), thinness (˂−2 SD); chi-square = 98.12. Boys: FM—low (˂13%), normal (13–23%), high (˃23%); MM—low (˂73%), normal (37–83%), high (˃83%). Girls: FM—(low (˂16%), normal (16–29%), high (˃29%); MM—low (˂67%), normal (67–80%), high (˃80%). FM: chi-square = 29.73; MM: chi-square = 27.36.

**Table 4:** Anthropometric measurements of the children (*n* = 66) with CD following the Ministry of Health GFD program according to a body composition analyzer.

Anthropometric Index	Boys (*n* = 29)	Girls (*n* = 37)	*t*-Test	*p*-Value
Weight (kg)	31.79 ± 16.76	41.57 ± 18.08	−2.25 *	0.028
Height (cm)	133.52 ± 21.38	143.70 ± 14.77	−2.19 *	0.034
BMI (kg/m^2^)	16.61 ± 3.79	19.63 ± 5.57	−2.61 *	0.011
FM (kg)	6.56 ± 5.58	13.38 ± 10.77	−3.32 **	0.002
FFM (kg)	25.23 ± 11.83	28.20 ± 8.27	−1.15	0.257
MM (kg)	23.78 ± 11.21	26.65 ± 7.87	−1.17	0.248
SMM (kg)	13.80 ± 6.90	15.50 ± 4.98	−1.11	0.271
FM (%)	18.79 ± 6.64	28.68 ± 9.30	−4.84 **	0.000
FFM (%)	81.20 ± 6.65	71.32 ± 9.30	4.83 **	0.000
MM (%)	76.45 ± 6.25	67.38 ± 8.76	4.71 **	0.000
SMM (%)	43.82 ± 4.53	38.74 ± 5.59	3.98 **	0.000
FMI (kg/m^2^)	3.31 ± 1.94	6.05 ± 3.96	−3.68 **	0.001
FFMI (kg/m^2^)	13.30 ± 2.11	13.33 ± 2.06	−0.06	0.951

Independent sample *t*-test; * *p* ≤ 0.05; ** *p* ≤ 0.01; SD—standard deviation; BMI—Body Mass Index; FM—Fat Mass; FFM—Fat Free Mass; MM—Muscle Mass; SMM–Skeletal Muscle Mass; FMI—Fat Mass Index; FFMI—Fat Free Mass Index; FM (kg) + FFM (kg) = weight (kg), (FMI (kg/m^2^) + FFMI (kg/m^2^) = BMI (kg/m^2^).

**Table 5:** Biochemical parameters in relation to reference range for children (*n* = 66) with CD following the Ministry of Health GFD program.

Variables	Boys (*n* = 29)		Girls (*n* = 37)		*t*-Test	*p*-Value *
	Mean ± SD	Ref. Range	Mean ± SD	Ref. Range		
Vitamin D (nmol/L)	42.11 ± 8.74	(75–350)	47.44 ± 13.76	(75–350)	−1.82 *	0.034
Hemoglobin (g/dL)	13.01 ± 1.47	(13.5–18)	13.00 ± 0.75	(11–15)	0.05	0.963
Albumin (g/L)	41.33 ± 8.05	(32–45)	42.23 ± 1.49	(38–54)	−0.66	0.509
Sodium (mmol/L)	138.10 ± 1.33	(136–145)	138.25 ± 1.37	(138–145)	−0.46	0.648
Potassium (mmol/L)	4.49 ± 0.25	(3.50–5.10)	4.34 ± 0.24	(3.40–4.70)	0.51	0.317
Alkaline Phosphatase (U/L)	244.25 ± 48.60	(89–365)	245.72 ± 56.58	(141–460)	−0.11	0.912
Calcium (mmol/L)	2.40 ± 0.06	(1.63–3.53)	2.39 ± 0.05	(2.22–2.70)	0.52	0.604

Independent sample *t*-test; * *p* ≤ 0.05.

**Table 6:** Spearman correlation coefficient and simple linear regression analysis between demographic data and some anthropometric indices of children (*n* = 66) with CD following the Ministry of Health GFD program for boys and girls.

Dependent Variable/Independent Variable	Boys (*n* = 29)						Girls (*n* = 37)					
	BMI		FM		MM		BMI		FM		MM	
	r	β, r^2^	r	β, r^2^	r	β, r^2^	r	β, r^2^	r	β, r^2^	r	β, r^2^
Age (years)	0.616 **	0.121 **, 0.380	0.686 **	0.083 **, 0.389	0.865 **	0.056 **, 0.708	0.509 **	0.063 **, 0.239	0.653 **	0.037 **, 0.308	0.710 **	0.066 **, 0.536
Father’s education	−0.259	−0.003, 0.0002	−0.314	−0.001, 0.00003	−0.205	−0.003, 0.003	−0.215	−0.022, 0.021	−0.146	−0.004, 0.003	−0.018	−0.003, 0.001
Mother’s education	−0.193	−0.024, 0.012	−0.224	−0.016, 0.012	−0.279	−0.019, 0.069	−0.184	−0.027, 0.027	−0.101	−0.012, 0.020	−0.058	−0.008, 0.004
Family’s education	0.265 *	0.120 *, 0.153	0.222 *	0.076 *, 0.135	0.334 *	0.040 *, 0.147	−0.166	−0.014, 0.004	−0.141	−0.003, 0.001	0.066	0.011, 0.005
Family members	0.367	0.077, 0.107	0.402 *	0.059 *, 0.136	0.460 *	0.038 *, 0.224	0.109	0.023, 0.035	0.144	0.014, 0.048	0.025	0.012, 0.019

* *p* ≤ 0.05; ** *p* ≤ 0.01; r = correlation coefficient; β = regression coefficient; partial r^2^ for independent variables of interest.

## Data Availability

The datasets used and analyzed during the current study are available from the corresponding author upon reasonable request.
